# Hemodynamic responses to emotional speech in two-month-old infants imaged using diffuse optical tomography

**DOI:** 10.1038/s41598-019-39993-7

**Published:** 2019-03-18

**Authors:** Shashank Shekhar, Ambika Maria, Kalle Kotilahti, Minna Huotilainen, Juha Heiskala, Jetro J. Tuulari, Pauliina Hirvi, Linnea Karlsson, Hasse Karlsson, Ilkka Nissilä

**Affiliations:** 1 University of Turku, Institute of Clinical Medicine, Turku Brain and Mind Center, FinnBrain Birth Cohort Study, Turku, Finland; 2 University of Mississippi Medical Center, Department of Neurology, Jackson, MS USA; 3 Department of Neuroscience and Biomedical Engineering, Aalto University, Helsinki, Finland; 4 CICERO Learning, Faculty of Educational Sciences, University of Helsinki, Helsinki, Finland; 5 Faculty of Educational Sciences, University of Helsinki, Helsinki, Finland; 6 Department of Clinical Neurophysiology, Helsinki University Central Hospital, Turku, Finland; 7 University of Turku and Turku University Hospital, Department of Child Psychiatry, Turku, Finland; 8 University of Turku and Turku University Hospital, Department of Psychiatry, Turku, Finland

## Abstract

Emotional speech is one of the principal forms of social communication in humans. In this study, we investigated neural processing of emotional speech (happy, angry, sad and neutral) in the left hemisphere of 21 two-month-old infants using diffuse optical tomography. Reconstructed total hemoglobin (HbT) images were analysed using adaptive voxel-based clustering and region-of-interest (ROI) analysis. We found a distributed happy > neutral response within the temporo-parietal cortex, peaking in the anterior temporal cortex; a negative HbT response to emotional speech (the average of the emotional speech conditions < baseline) in the temporo-parietal cortex, neutral > angry in the anterior superior temporal sulcus (STS), happy > angry in the superior temporal gyrus and posterior superior temporal sulcus, angry < baseline in the insula, superior temporal sulcus and superior temporal gyrus and happy < baseline in the anterior insula. These results suggest that left STS is more sensitive to happy speech as compared to angry speech, indicating that it might play an important role in processing positive emotions in two-month-old infants. Furthermore, happy speech (relative to neutral) seems to elicit more activation in the temporo-parietal cortex, thereby suggesting enhanced sensitivity of temporo-parietal cortex to positive emotional stimuli at this stage of infant development.

## Introduction

Emotion processing is a complex brain function and relies on connections between the limbic and non-limbic systems. Emotional responses in humans can be activated via innate mechanisms using recall memory or through different sensory inputs such as auditory^1,2^, visual^3,4^, tactile^5,6^, and olfactory^7,8^ either individually or in combination^9,10^. These stimuli, in turn, activate key areas of emotion processing centers in the subcortical areas, e.g., the amygdala, thalamus, hypothalamus, cingulate gyrus, hippocampus^11^, as well as in the cerebral cortex including the orbitofrontal cortex, prefrontal cortex, temporal cortex and occipital cortex^12–15^.

Emotional speech is one of the principal forms of social communication in humans^16^. The processing of emotions conveyed by speech is particularly important for infants as it helps them to discriminate between emotions and selectively respond to them^17^. The left hemispheric dominance to speech is found in young children^18–20^ and adults^21^. The earliest report of activation of left temporal areas in response to speech has been reported in neonates as early as 2–9 days of age^22,23^. Speech has many components and one of the components is prosody. The prosody of emotional speech refers to the patterns of stress and intonation and comprises a variable mixture of pitch, loudness, timbre, the rate of speech, and pauses. The processing of emotional prosody is seen to develop before the semantic component of speech^24^. Some studies report left hemispheric activation to speech^18,19,22^, while others report right hemispheric activation in response to speech prosodies^1,25–28^. In Kotilahti *et al*.^20^, no statistically significant lateralization was found to speech in neonates; however, at group level, there was a significant positive HbT response to speech on the left hemisphere only^20^. Thus, the hemispheric lateralization of speech would not appear to be consistent across subjects and studies in infancy.

Emotional speech processing has been increasingly investigated with various neuroimaging techniques such as electroencephalography (EEG), functional magnetic resonance imaging (fMRI) and near-infrared spectroscopy (NIRS). EEG has been widely used to study emotional processing in neonates and infants due to its high temporal resolution and good infant tolerance. Early emotional mismatch response shows a right lateralized pattern in 1- to 5-day-old neonates in response to happy valenced syllables vs. non-vocal sounds^29^. By the age of 7 months, there are different responses between the two hemispheres to happy and angry valenced prosody^17^. This suggests that there is an evolution of emotional processing during infancy and the brain responses to different emotional speech stimuli. Although evoked response potential (ERP) studies have provided important insights to emotional speech processing, they are limited in spatial resolution.

Studies using fMRI, which has a better spatial resolution than EEG, have shown that by the age of two months, there might be a left temporal activation to speech^18,19^ and right planum temporale activation to music^18^. However, fMRI studies are limited in newborns and infants due to technical challenges in data acquisition. For example, there is difficulty in isolating the scanner noise, maintaining infants’ prolonged sleep duration inside the tube, sensitivity to motion^30^, and the variability of the blood-oxygen-level dependent (BOLD) signal between various stimulus modalities^31^.

Functional near-infrared spectroscopy (fNIRS) has been increasingly used to study emotional speech processing in infants. Right temporal activation has been observed by Zhang *et al*. in response to emotional prosody, relative to neutral prosody, in neonates as early as 2–8 days after birth. Moreover, the researchers observed heightened sensitivity in a right parietal area (approximately located in the supramarginal gyrus) to fearful, relative to happy and neutral, prosody^28^. Right temporal activation has also been reported in response to emotional human voices (as compared to non-emotional voices) in 4-month-old infants^32^. Grossmann *et al*. noted specific angry > happy and angry > neutral differences in the right hemisphere^1^. Together, these findings support the role of right hemisphere in emotional speech processing in infants.

However, recent studies have also indicated the involvement of left hemisphere in speech processing in infants. An fMRI study by Blasi *et al*. reported a positive BOLD response in the bilateral middle temporal gyrus in response to human vocalizations in 3–7-month-old infants. Furthermore, the researchers observed a significantly more positive response to sad vocalizations than to emotionally neutral vocalizations in the left orbitofrontal cortex and insula^16^. Graham *et al*. reported left hemispheric positive BOLD responses to happy valenced semantically meaningless sentences in sleeping infants^33^. Infant-directed speech (as compared to reverse speech or silence) is seen to activate bilateral frontotemporal, frontal, temporal, and temporoparietal regions in the infants^28,34–38^. Human voice reportedly causes activation in the voice-selective regions of the bilateral temporal cortices in infants between 4 and 7 months of age^39^. Kotilahti *et al*. reported a statistically significant speech response at the group level only on the left hemisphere, although inter-subject differences were comparable in magnitude to inter-hemispheric differences^20^. Thus, altogether these findings indicate that the current literature does not show a clear hemispheric predominance to speech or emotion processing in infants^40^, although individual studies show aspects of speech and emotion processing in some cases statistically significant on one hemisphere but not in the other.

Diffuse optical tomography (DOT) is a three-dimensional imaging method that uses near-infrared light to obtain the optical properties of tissue^41–44^. DOT can measure changes in the concentrations of oxygenated (HbO_2_), deoxygenated hemoglobin (HbR) and total hemoglobin (HbT)^42,43^. The resolution of the diffuse optical imaging technology can be quite good (~1 cm). However, to realize its full potential, instruments which permit measuring many partially overlapping source-detector pairs with a range of separations are needed in combination with accurate modeling of light propagation in tissue and 3D image reconstruction methods, enhancing the spatial resolution and spatial and quantitative accuracy^45–49^. This technology is called high-density diffuse optical tomography (HD-DOT) and was first applied to the adult visual cortex by Zeff *et al*. in^47^. HD-DOT is a portable and quiet neuroimaging tool that is safe and easy to use and is generally well tolerated by infants ^43,48,49^, but the number of fibers used in infant studies, so far, has been limited by practical considerations^5^.

To establish a baseline of responses to emotional speech in two month-old infants with a new method (HD-DOT), we looked at the response averages across subjects and identified statistically significant features in the responses to emotional speech using three analysis methods: global analysis, voxel-based clustering analysis and region-of-interest (ROI) based-analysis. Our hypotheses were (1) emotional speech activates the auditory regions in the temporal cortex and (2) different response patterns can be identified for differentially valenced emotional speech stimuli in the left temporal cortex.

## Results

### Overview of the responses

**Figure 1:**
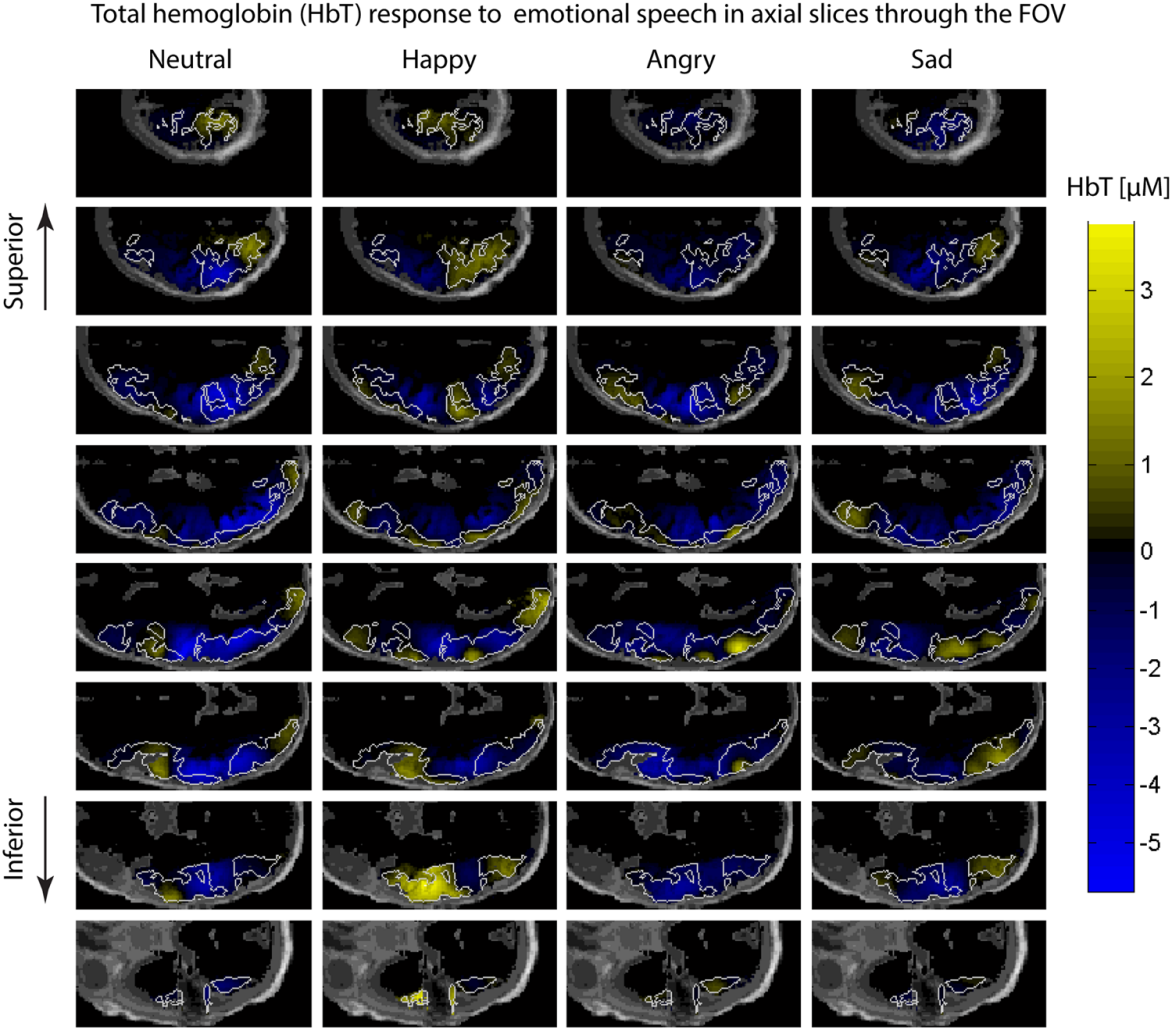
Axial slices of the left hemisphere showing the grand average responses over the 21 subjects within the time window [2 s, 18 s] from stimulus onset from top of the head to bottom in 10 mm intervals. Warm colors (yellow) indicate an increase in total hemoglobin (HbT) and cool colors (blue) indicate a decrease in HbT in response to the stimulus averaged in the 2 s to 18 s post stimulus onset time window. The scalp and skull are shown in dark gray. White line surrounds gray matter voxels within the field of view which are positive at least for one stimulus condition.

The spatial distributions of HbT responses averaged over the 21 subjects in the time interval from 2 s to 18 s after stimulus onset are shown in Fig. 1; each stimulus condition (emotion of speech) is displayed as a column, and each row shows axial slices from top of the head to the bottom at 10 mm intervals. Positive HbT responses are shown in yellow/orange and negative HbT responses in blue. Regions that had positive HbT responses to at least one stimulus condition are marked with a white outline.

### Global responses

**Figure 2:**
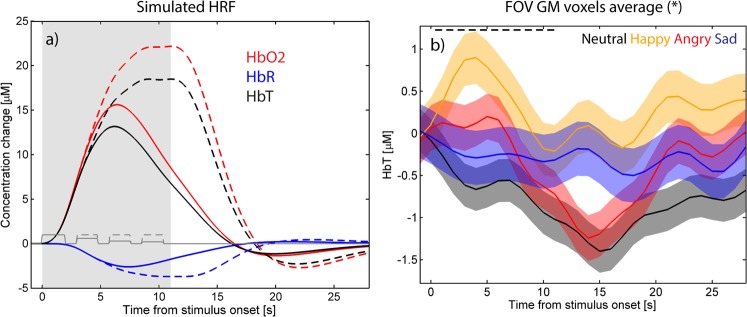
(**a**) The simulated shape of the hemodynamic response (HbR in blue, HbO_2_ in red, and HbT in black) obtained by convolving a square wave depicting the stimulus train consisting of four sentences and the adult canonical hemodynamic response function (HRF). Default parameters for the HRF were used for HbR, and the delay of onset of the response and undershoot for HbO_2_ were set to −1 s relative to corresponding parameters for the HbR. The ratio of HbR and HbO_2_ amplitudes was set to −1:6. HbT was obtained as a sum of HbO_2_ and HbR. The stimulus epoch is shaded in gray and the individual stimuli indicated with a dark gray line, with habituation after the first stimulus of a train factored into the amplitude (solid lines) and no habituation (dashed ‘--’ lines). (**b**) Time course of HbT responses to each of the four stimulus conditions (black = neutral, orange = happy, red = angry, and blue = sad speech) in the GM voxels of the region showing activation for at least one stimulus condition. Shading indicates standard error mean (SEM). Black dashed line (‘--’) indicates the time of stimulus presentation.

Simulated time courses based on the adult canonical hemodynamic response function and two habituation scenarios are shown in Fig. 2a (see Materials and Methods for details) for comparison purposes. The time courses of the measured HbT responses for each of the four speech stimuli (happy, angry, sad and neutral) were averaged over the gray matter (GM) voxels within the field-of-view (FOV) excluding the voxels that were negative for all  of the four emotional speech conditions (region shown outlined in Fig. 1; time courses in Fig. 2b). The means of the HbT responses within the 2 s to 18 s post-stimulus onset time window are shown in Table 1. The response to neutral speech was negative and statistically significant (neutral < baseline; Table 1). Analysis of variance (ANOVA) and Tukey-Kramer post hoc test revealed that the response to happy speech was significantly greater than the response to neutral speech (p = 0.01; Table 1). Interestingly, the time-to-peak for the response to happy speech was only 4 s, suggesting strong habituation after the first stimulus of the four-stimulus train, whereas the negative responses to neutral and angry condition peaked at 14 to 15 s post stimulus onset.

**Table 1:** HbT response mean values averaged across the 21 subjects within the time window from 2s to 18s and p-values indicating statistical significance of the difference between the response and baseline, evaluated using two-tailed Student’s t test for cluster C1 which shows a statistically significant negative mean speech response averaged across the conditions, for cluster C2 which shows a significant negative response to neutral speech.

	HbT 2–18 s	p uncorr/corr. Two-tailed t test
GM voxels with HbT > 0 for at least one stimulus condition averaged in the FOV (Figs 1 and 2b) N_vox_ = 28191		N_MC_ = 1 ANOVA: p = 0.011** post hoc: happy > neutral**
Speech Average	−0.3	0.39
Neutral	−1.0	6.1 × 10^−4^
Happy	0.5	0.21
Angry	−0.6	0.055
Sad	−0.3	0.48
C1 Temporal cortex (Fig. 3a–c) Speech average < baseline N_vox_ = 792 (L3)		N_MC_ = 120 ANOVA p = 0.64
Speech Average	−2.5**	3.3 × 10^−4^/0.04**
Neutral	−3.7*	0.037*/−
Happy	−0.8	0.65/−
Angry	−3	0.084/−
Sad	−2.6	0.13/−
C2 Temporal cortex (Fig. 3d–f) Neutral < baseline N_vox_ = 471 (L2)		N_MC_ = 120 ANOVA p = 0.020* Angry > neutral*
Speech Average	−1.5*	5.3 × 10^−3^*
Neutral	−4.1**	7.0 × 10^−6^/8.4 × 10^−4^**
Happy	−1.2	0.32/−
Angry	−0.2	0.82/−
Sad	−0.6	0.50/−

The p-values for the clusters are given both as uncorrected values as well as corrected for multiple comparisons using Bonferroni (N = 120). For clusters C1-C2, the voxel-wise significance level that gives the optimal cluster-wise significance is stated (L1 p_th_ = 0.001; L2 p_th_ = 0.0033; L3 p_th_ = 0.01) along with the cluster extent. *p < 0.05 uncorrected; **p < 0.05 corrected for the number of regions using Bonferroni method (N = 1 for the global test, N = 120 for clusters).

### Results from voxel-based clustering

#### Regions showing responses to emotional speech

**Figure 3:**
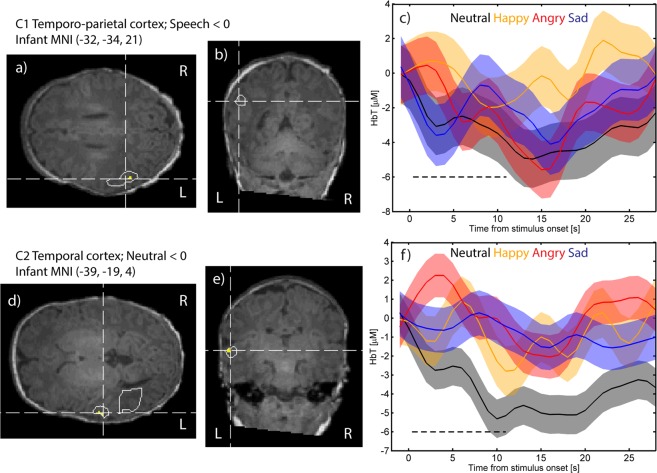
Axial and coronal slices for clusters C1-C2, with voxels that satisfy p < 0.001 marked in yellow and p = 0.01 marked with white contour line, and the corresponding HbT response time courses for each stimulus condition. C1 shows a significant negative response to speech (**a**–**c**); C2 a significant negative response to neutral speech (**d**–**f**). Neutral speech is marked in black, happy speech in orange, angry speech in red and sad speech in blue. The shaded area shows the standard error mean (SEM).

A statistically significant negative HbT response to emotional speech (the average of the emotional speech conditions < baseline) was found in the left temporo-parietal cortex (Cluster 1 (C1); Fig. 3a,b) averaged over all conditions and 21 infants. The time course for the responses to each condition are given in Fig. 3c. Mean responses and p-values for the two-time windows are given in Table 1.

#### Regions showing statistically significant responses to one or more stimulus condition(s)

Neutral speech: Statistically significant negative responses to neutral speech (neutral < baseline) were found in one cluster (C2) in the temporal cortex shown in Fig. 3d–e (negative). The corresponding time courses are shown in Fig. 3f. The response magnitudes and p-values are shown in Table 1.

Happy, angry and sad speech: No clusters were found using the voxel-level clustering technique where the response to happy, angry, or sad speech was statistically significantly different from baseline.

#### Regions showing emotion-specific responses

No regions showing statistically significant differences between emotion-specific stimuli in the ANOVA test with multiple comparison corrections were  found using voxel-level clustering.

### Results from ROI-based analyis

**Figure 4:**
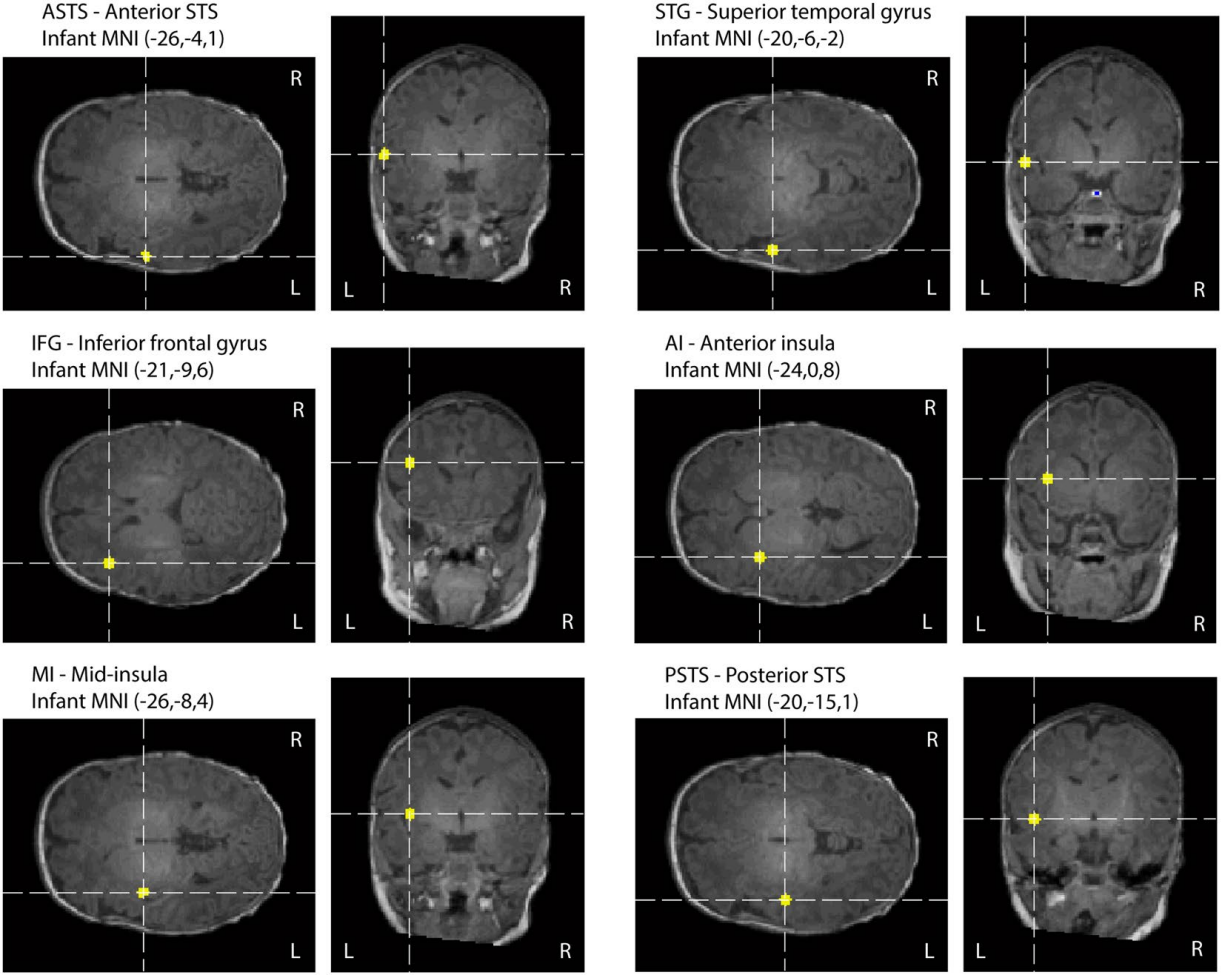
Locations of the regions of interest (ROIs) used in this study.

Analysis of variance (ANOVA) followed by Tukey-Kramer post hoc test found that neutral > angry in the anterior superior temporal sulcus (aSTS) ROI; happy > angry in the left Superior Temporal Gyrus (STG) ROI, happy > angry and happy > neutral in the posterior STS (pSTS) ROI (Table 2; Fig. 4). Additionally, the responses in each of the ROIs were compared with baseline (BL) using two-way Student’s t-test. We found a negative HbT response to angry (angry < baseline) in the anterior and posterior STS (aSTS and pSTS ROIs, respectively), in the STG ROI and in anterior and mid-insula (AI and MI ROIs) (Table 2; Fig. 4). Finally, in the anterior insula (AI) ROI, happy < baseline was statistically significant. Time courses for the responses to each of the emotional speech conditions in the ROIs are shown in the supplement.

**Table 2:** ROIs, their approximate infant MNI coordinates, HbT responses for each speech condition averaged over a time window of 2 to 18 seconds, statistical significance of the difference between conditions based on ANOVA and Tukey-Kramer post hoc test (first data column) and statistical significance of the response vs. baseline (BL) based on two-way Student’s t-test.

	Approx. infant MNI coordinates	ANOVA p-value	Neutral HbT	Neutral p-value	Happy HbT	Happy p-value	Angry HbT	Angry p-value	Sad HbT	Sad p-value
aSTS	(−26, −4, 1)	0.036* neutral > angry*	1	0.42	0.6	0.61	−3.2	7.3 × 10^−3^** angry < BL**	−1.1	0.28
STG	(−20, −6, −2)	0.025* happy > angry*	0.4	0.74	1	0.42	−3.8	0.013* angry < <BL*	−0.6	0.52
IFG	(−21, −9, 6)	0.56	−0.5	0.059	−0.25	0.073	0	0.79	−0.3	0.07
AI	(−24, 0, 8)	0.29	0.42	0.68	−0.8	0.048* happy < BL*	−2	0.012* angry < BL*	−1.3	0.2
MI	(−26, −8, 4)	0.062	1	0.4	0.4	0.72	−2.9	9.2 × 10^−3^* angry < BL*	−1.2	0.28
pSTS	(−20, −15, 1)	0.021* happy > angry*; happy > neutral*	0.9	0.48	8.5	0.48	−3.6	7.5 × 10^−3**^ angry < BL^**^	−0.9	0.32

*p < 0.05 uncorrected; **p < 0.05 corrected for multiple comparisons for six regions. The coordinates are based on the UNC infant 0-1-2 template (Shi *et al*.)^102^. aSTS: Anterior superior temporal sulcus, STG: Superior temporal gyrus, IFG: Inferior frontal gyrus, AI: Anterior insula, PI: Posterior insula and pSTS: Posterior superior temporal sulcus.

## Discussion

In our current study, we investigated total hemoglobin (HbT) responses to emotional speech stimuli in the left frontotemporal cortex of two-month-old infants using diffuse optical tomography (DOT). Typically, an increase in local synaptic activity in the brain elicits positive responses in HbT and HbO_2_, a negative response in HbR and a positive response in the fMRI blood oxygen-level-dependent (BOLD) signal. Thus, brain activation-elicited HbT and BOLD responses are both positive. In infants, atypical responses are usually seen as inverted responses, i.e., negative HbO_2_ and HbT responses and positive HbR, and have been reported in the temporal cortex in response to auditory stimuli^50^.

First, we investigated whether responses to the different emotional speech stimuli (neutral, happy, angry and sad) elicited differential responses using analysis of variance (ANOVA) and post-hoc testing. In the global analysis of the FOV, excluding regions which produced negative responses to all four stimulus types, we found the HbT response to happy speech to be greater than the response to neutral speech.  The location of the response is distributed across the temporal and parietal cortices, with the largest contrast occurring in the anterior part of the temporal cortex. In line with our happy > neutral finding, a NIRS study by Zhang *et al*. also reported significant activation in the left IFG and frontal eye field to happy prosody contrasted with fearful and angry prosodies in adults^51^. A recent fMRI study by Koelsch *et al*. that compared responses to joy- and fear-inducing as well as neutral musical stimuli in adults, reported a statistically significant joy > neutral response in the left planum polare (as well as in the right planum temporale and left calcarine sulcus)^52^. Another fMRI study by Graham *et al*. also observed left hemispheric positive BOLD responses to happy valenced semantically meaningless sentences in sleeping infants. The authors reported that the happy tone of voice resulted in greater response than neutral in the left lingual gyrus, fusiform, parahippocampal gyrus, putamen, midcingulate, supplementary motor area, superior frontal gyrus and medial frontal gyrus^33^.

In contrast, a few studies compared the hemispheric responses to varied prosody and noticed a right hemispheric predominant involvement in processing emotional prosody. For example, emotional prosody has been seen to modulate responses in right temporal areas in neonates^28^ and right inferior frontal cortex in seven-month-old infants^17^. Analyzing the whole brain using fMRI, the adult brain showed bi-hemispheric activation (STG) responses to both positive and negative emotional stimuli compared to emotionally neutral sound^53,54^ but more so in the right hemisphere^55,56^. Kotz *et al*. observed increased activation in the right IFG in response to happy vs. neutral prosodies in adults^55^. Grossmann *et al*. reported negative HbO_2_ responses to neutral sounds in areas of the left temporal cortex with happy > angry > neutral sounds in 5-month old infants, although the differences in the left hemisphere were not statistically significant^1^. These results suggest that the left hemisphere plays an important part in the processing of emotional components but future studies are needed to understand the precise role of left and right hemispheres in response to emotional valences of speech in infancy.

By considering our literature-based regions of interest (ROIs), and continuing with the ANOVA and post-hoc analysis approach, we found happy > angry in the posterior superior temporal sulcus (pSTS) and superior temporal gyrus (STG). We found that happy speech elicited a greater response in the posterior STS than neutral speech, while neutral speech elicited a greater response in the anterior STS than angry speech. In line with our finding, Koelsch *et al*. reported greater response to neutral than fear-inducing music in the planum temporale in both hemispheres^52^. By contrast, Grandjean *et al*. found angry speech to elicit a greater BOLD response than neutral speech in the middle part of the right and left STS in adult subjects^57^; although in our results, areas in the middle STS show angry > neutral (Fig. 1), they are not statistically significant. Johnstone *et al*. reported greater BOLD response in anterior and mid-insula for happy than angry voice in adult subjects^58^, consistent with our present infant data.

Previous studies have implicated STG in the processing of language and components of prosody^53,54,59,60^. Koelsch *et al*. reported adult BOLD responses to joy-inducing music to be greater than fear-inducing music in the left (and right) transverse temporal gyrus^52^. Using balanced speech and functional fMRI in adult subjects, Wittfoth and colleagues showed STG activation for happily intonated sentences expressing a negative content^61^. In an fMRI study in 3- to 7-month-old infants, Blasi *et al*. reported that the age of infants positively correlated with the contrast between neutral voice vs. non-voice in the left STG^16^. They reported a significant sad > neutral finding in the left insula and gyrus rectus; our data is consistent with this finding regarding the insula, although not statistically significant.

Using clustering based on voxel-level statistics, we found statistically significant negative HbT responses to neutral speech and speech average clusters in the temporo-parietal cortex. The negative responses may be a result of the attenuation of the normal functionality of the resting-state activity in the brain due to the temporary reallocation of resources during the performance of a task^62–64^. In line with our negative HbT response findings, Grossmann *et al*. also reported negative HbO_2_ responses to neutral sounds in areas of the left temporal cortex with happy > angry > neutral sounds in 5-month old infants^1^. Similarly, Gomez *et al*. reported a negative HbO_2_ response to well-formed syllables in the left and right frontoparietal and temporal-perisylvian regions in newborn infants^65^. Bauernfeind *et al*. observed negative HbO_2_ responses in the central and parietal regions of both hemispheres and positive HbO_2_ responses in the middle temporal and frontal cortices in adults when exposed to pure tone auditory stimuli^66^. Additionally, we observed angry < baseline in aSTS, pSTS, STG, anterior insula and mid-insula, as well as happy < baseline in the anterior insula.

The observed time courses of the responses to emotional speech (e.g., Fig. 2b) do not perfectly match the canonically predicted hemodynamic response (Fig. 2a, illustrating the expected response in the habituated and non-habituated cases) in the present study. Infant hemodynamic responses are often reported to take longer to peak and return to baseline than adult hemodynamic responses^50,67,68^. The positive response to happy speech in the present study is relatively short in duration with peak response at 4–5 s and return to baseline at around 10 s post stimulus train onset. This suggests strong habituation within the stimulus block after the first spoken phrase. The negative responses to angry and neutral speech follow a longer time course with time to peak being at 14–15 s post stimulus onset.

Issard and Gervain have reviewed the variability of infant hemodynamic responses in the NIRS literature, including canonical (HbO_2_ and HbT increase, HbR decrease) and inverted responses (either HbO_2_ and HbT decrease and HbR increases, or all three parameters increase in response to stimulus)^50^. Many auditory studies in infants show canonical responses for HbO_2_, however, there is greater variability in the sign of HbR across subjects and stimulus conditions in the published literature^69,70^. Negative or flat HbO_2_ responses are reported in response to irrelevant (speaking the name of another person than the subject)^71^, or rapidly repeated stimuli of the same type^68^, which suggests neuronal deactivation or habituation, respectively. Habituation allows the brain to focus its processing on novel information by suppressing repetitive stimuli. Whether the inverted response is due to the immaturity of the neurovascular coupling or differences in neuronal processing is not clear. Issard and Gervain noted that infants develop canonical responses to social stimuli earlier than non-social stimuli^68^. This may support the idea that the infant brain prioritizes processing of stimuli that are relevant at that point in development over stimuli that are not especially relevant. More positive HbT responses to happy rather than neutral speech may differentiate infant-directed speech from adult-to-adult communication and may assist in bonding with the parent.

Possible explanations to negative HbT responses include deactivation, i.e. reduction of synaptic activity within the region in response to stimulus^72^, or alternatively a purely vascular effect; when there is stimulus-elicited activation and positive HbT response in one region, surrounding areas may observe reduction of blood pressure, cerebral blood flow and volume^72–75^. Boorman *et al*. combined fMRI, optical imaging spectroscopy, laser doppler flowmetry and electrode recordings to find negative BOLD responses, increased HbR and decreased blood flow in areas of the rat somatosensory cortex which showed decreased neuronal activation^76^. Negative responses to neutral speech, are reported bilaterally in Grossmann *et al*. in 4- to 7-month-old infants^1^ and Bauernfeind *et al*. in adults^66^ in response to bilateral sounds. However, we observed no corresponding positive responses to neutral speech within the FOV of our probe that could explain it as a blood stealing effect.

Sleep stage is known to affect hemodynamic responses in infants; Kotilahti *et al*. reported that auditory HbO_2_ responses in newborn infants were larger in active than in quiet sleep^67^. In adults, negative BOLD is observed in the primary auditory cortex^77^, or both in the auditory and visual cortices^78^ during sleep. These negative responses are more diffuse compared to positive responses observed during the awake state and may involve the secondary auditory cortex^79^. In the present study, we did not record EEG and the video quality was only sufficient to detect subject movement and not sleep/awake status due to the dim lighting in the room and wide angle of view of the video. A priority was given to include the mother in the video in order to study mother-child interaction and to monitor other events that might be going on in the room, so a wide angle of view was chosen. In order to examine a possible correlation between the mother’s spontaneous reactions to emotional speech stimuli with infant neural responses to emotional speech, we find the present data does not contain sufficient repetitions of each stimulus type to analyze the interaction in a reliable way. In future studies with higher-quality video recordings, ideally from multiple angles, with a larger number of stimulus repetitions, this kind of analysis may be possible. In our previous study on two-month old infants, the subjects were asleep approximately 50% of the time during the recording, and since the motion artifacts were more prevalent in awake state, 70% of the responses retained for averaging were presented during sleep^5^. The effect of sleep on the responses could be studied with higher-quality video recording and EEG, although attaching electrodes can contribute to subject discomfort. In practice, we found that obtaining high-quality awake data of young infants was challenging.

Methodologically, DOT appears to be well suited to auditory studies in infants as it is a virtually silent method that permits measurements where the infant is cradled in his or her mother’s lap. Background physiology is a frequently discussed topic in fNIRS and DOT studies. In infants, the scalp and skull are relatively thin, and the brain tissue starts at approximately 5 mm from the outer surface of the scalp, so the contribution of the superficial tissue to the recorded signals is smaller than in adults. In this study, we used superficial signal regression (SSR) and 3D image reconstruction to separate scalp physiology and global physiology from brain responses. The effect of SSR was visible in the averaged signals, reducing the response magnitudes in some cases, but the results calculated from reconstructed images were visually very similar with and without SSR. For brevity, we only presented the results from analysis where the SSR step was included, as the p-values were slightly smaller in some cases than in the results from the analysis without SSR. We think that this processing step is useful when the stimulus causes a strong-coupled physiological effect but we generally recommend using stimuli that do not cause strong autonomous nervous system responses. We removed the epochs of the data where the infant was either crying or moving vigorously, or if there was head movement, to avoid artifacts in the responses. In future studies, a shorter stimulus block would permit a greater number of repetitions and thus, likely, greater contrast-to-noise ratio in the results, and it is unlikely that there would be significant drawbacks especially given the strong habituation observed in the response to happy speech. The source power can be increased significantly as well, reducing the effects of photon noise and leading to a greater sensitivity to deeper tissues. HD-DOT has a drawback that there are practical difficulties in obtaining whole-head coverage in infant studies with current fiberoptic probe technology, since increasing the number of fibers can lead to an increased risk of interruptions in the measurement. Wireless technology with custom-made integrated circuits may make it easier to record NIRS and DOT data on children in the future, because the optical fibers limit the subject movement and reduce subject comfort.

## Conclusions

Our results show that total hemodynamic responses to emotional speech in two-month-old infants are in many ways similar to corresponding adult responses, including a distributed temporo-parietal happy > neutral response peaking the anterior part of the temporal cortex, happy > angry in the posterior superior temporal sulcus and superior temporal gyrus, as well as a negative total hemoglobin response to speech in medial and posterior temporal cortex. Our findings suggest that using HD-DOT for studying the emotional speech processing in infants provides interesting new information about infant brain development. Our results indicate that the infant left temporo-parietal cortex is preferentially activated by happy rather than neutral speech, which could imply preferential processing of the more relevant stimuli at that stage of development. Finally, our findings highlight the crucial role of left superior temporal sulcus in processing more positive emotions, such as happy speech compared to angry speech in two-month-old infants.

## Materials and Methods

### Study Design and Participants

The study population consisted of 21 infants (9 female and 12 male) that were born between June 2012 and October 2014, to mothers participating in the FinnBrain Birth Cohort Study^80^. The Joint Ethics Committee of the University of Turku and the Hospital District of Southwest Finland approved the study protocol, and all the methods used in the study were consistent with the approved protocol. The study was conducted according to the declaration of Helsinki. Parents provided written informed consent on behalf of the participating infants and were informed that they could cancel their participation in the study at any time.

The background information on the maternal due date of delivery was obtained when the mothers were recruited in the FinnBrain Birth Cohort Study at gestational week 12. The information on maternal age at birth and infant birth weight, height and head circumference were collected from the Medical Birth Register, National Institute for Health and Welfare (https://www.thl.fi/en/web/thlfi-en/statistics/information-on-statistics/register-descriptions/newborns). The descriptive statistics of the participating infants are given in Table 3. The age of the infants ranged from 6 to 10 weeks (mean ± SD 55 ± 9 days). Of the 46 infants that came for the measurements, recordings from 25 (54%) did not include a sufficient number of artifact-free repetitions of each stimulus condition (>=5), and the analysis was based on the remaining 21 (46%) infants. This matches the median attrition rate of 54% in NIRS infant studies with greater than 20 optodes in the literature^81^.

**Table 3:** Descriptive statistics of the participant infants (N = 21).

	Median	Range
Age at measurement calculated from term (days)	54.0	27–74
Age at measurement calculated from the birthdate (days)	52.0	43–71
Gestational weeks at birth	39.9	37–42
Head circumference at birth (cm)	38.5	33–42
Birth weight (g)	3500.0	2525–4175
Birth height (cm)	51.0	47–54
Maternal age at measurement (years)	32.1	21–37

### Instrumentation

We used a diffuse optical tomography (DOT) system built at Aalto University^82,83^ in this study. We chose to measure HbT only because of the following reasons: Synaptic activity modulates HbT through arterial dilation. Oxygen metabolism converts HbO_2_ into HbR and does not directly affect HbT. Both arterial dilation and oxygen metabolism affect HbO_2_ and HbR, but typically in opposite directions: if synaptic activity increases, HbO_2_ is increased by arterial dilation and decreased by metabolism, and HbR is decreased by arterial dilation (HbR is flushed out) and increased by metabolism, so the two effects pull these parameters in opposite directions, thereby potentially causing non-monotonous relationship between neuronal activity and hemodynamic parameters. In our previous studies^5,20,83^, we found the best statistical significance between conditions using HbT. In the literature^84^, HbO_2_ is more commonly reported, likely because the contrast is higher, but HbT and HbO_2_ responses are in practice quite similar to each other. HbT and HbO_2_ divergence can occur when the stimulus presentation rate is changed, but in the current study, the stimuli were presented at a normal rate of adult speech. Of the successful measurements, 19 were recorded with 798 nm and two were recorded with a pair of 758 nm and 824 nm temperature stabilized laser diodes. The wavelengths were measured with a calibrated spectrometer. The effect of the different wavelength configurations between subjects and uncertainty in the extinction coefficients was estimated to potentially cause an error up to ~1.5% in the grand average results.

Microelectromechanical system (MEMS) technology switches (Opneti Ltd., China) were used to switch between source fibers and wavelengths. Silicone-based high-density fiberoptic probes (Accutrans, Ultronics/Coltène) with 15 source fibers and 15 detector fiber bundles were used with a self-adhesive bandage to attach the probe on the subject’s head. The source positions were activated sequentially and the high voltage of each detector was adjusted to optimize the signal quality for each source-detector pair. The image frame rate was approximately 1.2 s.

### Procedure

**Figure 5:**
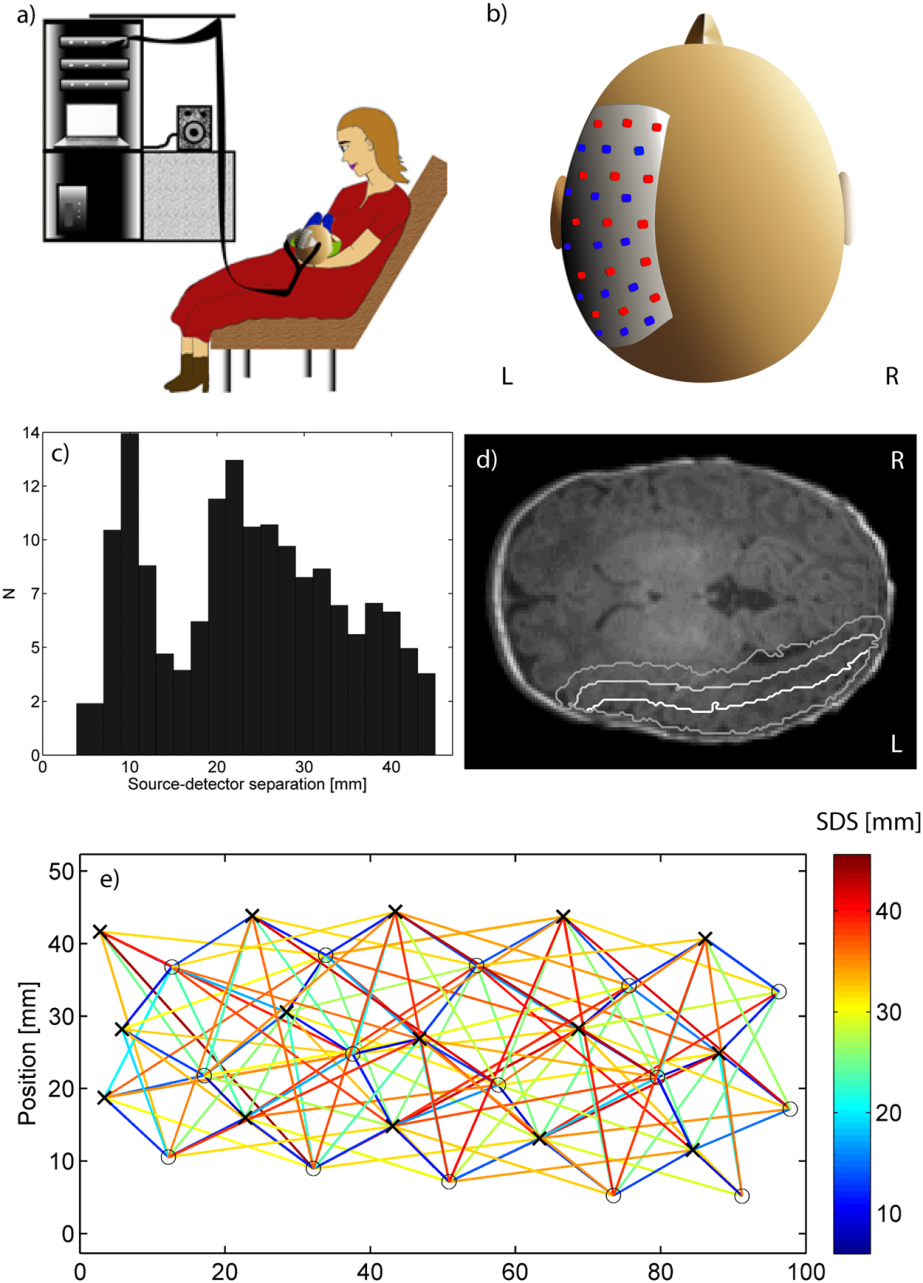
(**a**) Illustration of the measurement session (drawn by author SS). (**b**) Position of the probe on the subject’s head. (**c**) Histogram of source-detector separations used in the imaging. (**d**) The relative sensitivity map thresholded at 0.1 (white line), 0.01 (light gray line) and 0.001 (dark gray line) superimposed on an axial slice. (**e**) Relative positions of the sources (black crosses) and detectors (gray circles) with color-coding of the interconnecting lines indicating source-detector distances (SDS) up to 45 mm.

During the session, the mother was sitting on a comfortable chair and the infant was lying on the mother’s lap to provide a safe and comfortable environment for the infant (Fig. 5a). Before the recording, the mother was encouraged to breastfeed the infant to improve the likelihood of a peaceful recording but in some cases the mother also breastfed during the recording. The exclusion of data was based on infant movement as observed from the video recording and abrupt changes in the modulation amplitude signal. Breastfeeding periods were not excluded if there was no vigorous movement associated with it. Photogrammetry markers were placed on the infant’s face and head while they were sitting on their mother’s lap. A stereo camera setup was used to record images of the subject and markers from five to seven different directions. The measurement probe was then attached to the left temporal cortex using self-adhesive bandage wrapped around the subject’s head (Fig. 5b). After the probe was attached, additional stereo images were taken to record the position of the probe relative to the landmarks. The entire measurement session was recorded using a video camera. After the measurement session was started, if the infant was uncomfortable or crying, the measurement was paused to re-feed or console the infant. Once the infant was calm again, the mother was asked for permission to continue. If the infant continued to be uncomfortable, the measurement session was terminated. The duration of the measurement session including preparation time was approximately 1 h 30 min.

### Stimulation

The stimuli consisted of 11-second blocks of four short phrases (different content but the same emotion within each block) spoken in Finnish by an actress and presented using a computer running Presentation software (Neurobehavioral Systems, USA) and a loudspeaker. The sound intensity was set to approximately 65 dB. Happy, sad, angry and neutral emotional speech was used in this study. The rest period was randomized in duration from 20 s to 30 s between each block.

### Video analysis

The video recordings of each session were analyzed to determine the time points where audible external noise, head movement, limb movement or crying was present. Affected epochs were excluded from averaging. For our present study, 46 infants came to the measurement session, out of which 21 infants (9 females and 12 males) were successfully measured.

### Signal processing

The amplitude signals were first resampled to a common time base with 1 Hz sampling frequency using linear interpolation, to synchronize the data from different source positions. The signals were bandpass filtered with −3 dB cutoff frequencies of 0.007 Hz and 0.2 Hz to alleviate the effects of drift, contact variation and high-frequency noise on the signal. The lower frequency limit was selected to minimize the distortion of the hemodynamic response shape and the higher frequency to make the time course figures easier to read as well as to reduce the sensitivity to the precise selection of the time window used to determine the response magnitude. Superficial signal regression (SSR) was used with a regressor formed by averaging the source-detector (SD) pairs with separation lower than 12 mm to reduce the effects of global physiology on the estimated hemodynamic responses in the brain. The effect of SSR on the results was minimal. The distribution of Euclidian source-detector separations (SDSs) used is shown in Fig. 5c and the layout of sources and detectors is shown in Fig. 5e along with interconnecting lines indicating source-detector pairs with SDS <= 45 mm. The field of view (FOV) of the imaging probe was estimated based on the measurement sensitivity to absorption changes in the tissue underlying the probe. First, we calculated the normalized Jacobians for each source-detector pair by dividing the Jacobian with its maximum value within the brain tissue, and for each voxel, taking the maximum value of the normalized Jacobian over all source-detector pairs. The FOV was set to include all gray and white brain matter voxels for which the relative sensitivity was greater than 0.001 for at least seven subjects. The corresponding contour lines for thresholds (0.1, 0.01 and 0.001) are shown in a contour plot superimposed on an axial slice of the segmented MRI in Fig. 5d. The reconstructed contrast of absorption changes deep in the brain is lower than in superficial cortical tissue, for example, the HbT change in the insula reported in Jönsson *et al*.^5^ is about one-fifth in magnitude compared to the HbT change in the middle temporal gyrus^5^. However, it should be noted that the reconstructed contrast does not fall off as quickly as the measurement sensitivity does as a function of depth, since each source-detector pair influences the reconstruction with different weights. In this study, the phase signal was not used due to the low source power (mean 0.2 mW). In addition to the exclusion of stimuli based on the video recording, the absolute value of the high-pass filtered amplitude signal was compared with a threshold set to 3.5–7 times the standard deviation of the signal, and stimulus triggers corresponding to those periods where the amplitude exceeded the threshold were also excluded. The threshold was selected for each subject by visual evaluation of the signals and the L-curve method. The majority of signal epoch rejections were marked based on the observed movements in the video. Typically, the signal epochs marked for rejection based on the videos showed greater amplitude fluctuations than the signal during epochs while the infant was not moving. The observed levels of fluctuations during known movement periods were compared with the fluctuations in the rest of the signal and used to guide the selection of a rejection threshold, so that individual movement artifacts that were not marked in the video could still be rejected. The video coding was done by author SS and the visual inspection of data was done by author IN. Both steps were repeated to improve consistency of the evaluation across the data set. After signal processing, deconvolution was used to obtain estimates of the average responses to each stimulus type.

### Photogrammetry

Stereo photogrammetry images were captured from different sides of the subject’s head (five to seven images) with sticker markers (with black and white checkerboard pattern inside a circle) positioned on the nasion, left and right preauricular points (NAS, LPA, and RPA, respectively), on the cheeks and chin. The head was covered with a stretchable mesh with approximately 600 colored glass pearl markers at nodes in a regular 3 × 3 pattern for easier identification of matching points between pairs of stereo images and between image pairs taken from different directions. The 3D point coordinates of each node and marker were then determined relative to a fixed camera coordinate system, and the points were co-registered with an MR image of a representative infant from the FinnBrain MRI sub-study^6^ using LPA, NAS, and RPA. The probe and optode positions were determined from additional images that were made with the probe attached to the head. Distances between landmarks were measured from the photogrammetry and the mean, standard deviation and range for LPA-RPA were 99 mm ± 6 mm (range 92–106 mm), INI-NAS 137 mm ± 6 mm (range 123–146 mm), and head circumference (HC) 380 mm ± 17 mm (range 351–410 mm).

### Anatomical model

A voxel-based anatomical model was created by segmenting the T1 image of a representative healthy infant from the FinnBrain MRI sub-study^6^, with size and shape typical of the age group. Although a probabilistic atlas^85^ would have the benefit of greater accuracy across a population of subjects, in the fast-growing phase of infancy, the generation of accurate age-specific atlases including the superficial layers may not give sufficient benefits to warrant the extra work. The use of high-density DOT has the benefit of reducing the sensitivity of the method to optical parameter errors^46^. The anatomical image was segmented into tissue types with optical properties according to the values given in Jönsson *et al*.^5^. The voxel size in the original MR image was 1 mm × 1 mm × 1 mm. However, to account for the variation in head sizes between subjects, the voxel side length was scaled to minimize the squared Euclidian radial distance between the photogrammetry markers and the surface of the scalp in the scaled voxel model (resulting voxel side length in the model have mean ± SD 1.068 mm ± 0.025 mm). The coregistration of the anatomical image with the photogrammetry points was done using in-house software written in MATLAB. Monte Carlo simulation performed with Monte Carlo eXtreme software^86^ was used to calculate the spatial sensitivity of the amplitude measurement to changes in the absorption coefficient in a 2 × 2 × 2 voxel grid.

### Image reconstruction

A linear reconstruction method was used to estimate absorption changes inside the head model, and the absorption changes were converted into total hemoglobin concentration changes (HbT) using extinction coefficients using the Beer-Lambert law^87^. Laplacian smoothing regularization was used to reduce the noise in the images.

### Time course

Prior to image analysis and statistical testing, the pre-stimulus average within the window [−1, 0] s was subtracted from the time courses to establish a common baseline. Arichi *et al*. evaluated somatosensory fMRI BOLD time-to-peak values in infants as a function of postmenstrual age^88^ and Kotilahti *et al*. reported auditory NIRS HbO_2_ response time-to-peak values in term newborn infants as a function of gestational age^67^. Although at birth, the variability in response time-to-peak across subjects is larger than in adults^89^, the variability is expected to be reduced as the brain matures. In the present study, we used the adult canonical hemodynamic response function (HRF) as a guide to understand and interpret the time course of the hemodynamic response to a train of stimuli.

We expect that when presenting four short phrases in quick succession, with long pauses between trains, there is some habituation of the neuronal response to the second, third and last stimulus^89,90^. Figure 2a illustrates the time course of the four speech epochs with two potential neuronal habituation scenarios factored in; the dashed line corresponds to no habituation and the solid line to amplitudes 1, 0.6, 0.3, and 0.2. The hemodynamic response was estimated by convolving the canonical HRF with the habituated stimulus train (red, blue and black lines; solid line (‘-’) = habituation included, dashed line (‘- -’) = no habituation). The HRF was shifted by −1 s for HbO_2_ to account for the slightly faster response compared to HbR and BOLD. The HbR response was divided by −6 to obtain a HbR: HbO_2_ ratio of −1:6. Based on these graphs, we selected the time window of 2 s to 18 s from the onset of the stimulus train as the time window for which the hemodynamic response magnitude is estimated, which then is used for statistical testing at the group level. We also briefly explored the use of longer time windows (2–21 s and 2–24 s), but because of the high temporal correlation in the responses, the results are largely unchanged by the inclusion of additional seconds to the end of the time window. We found that there is greater variability across subjects in the return to baseline period than in the onset of the response^5^. This may be, partly, due to the subtraction of baseline, which controls the variability in the onset phase more effectively than in the return to baseline phase, but it may also be physiological in origin. In a few studies^84,91^, a positive HbR response is found in combination with a positive HbO_2_ response; for example, Wilcox *et al*. report positive HbO_2_ and negative HbR in the primary visual cortex in response to visual stimulus but positive HbO_2_ and positive HbR in the inferior temporal cortex in 6.5-month-old infants. However, the HbT parameter was consistently positive in both the primary visual cortex and the inferior temporal cortex^91^. The HbT average within the time window 2 to 18 s post stimulus onset subtracted by the mean of the pre-stimulus baseline interval [−1 s 0 s] was used as the measure of the magnitude of the hemodynamic response throughout the rest of the paper in the statistical testing.

### Image analysis and statistical testing

Our goal was to identify regions where there is a statistically significant speech response as well as regions which show statistically significantly different responses to the different emotional stimuli. We first started with global analysis and then proceeded to identify regions by using voxel-based statistics as a guide and merging similar, but adjacent voxels into clusters.

#### Global analysis

First, we wanted to consider the imaging field of view (FOV; region inside the 0.001 line in Fig. 5d) as a whole and investigate the response to speech and the differences between responses to the different types of emotional speech. This was done by averaging the HbT responses over all GM voxels within the FOV and over the time window from 2 s to 18 s from stimulus onset. We performed two types of statistical tests: (1) averaging the responses to all four emotional speech conditions and comparing the resulting average with zero using Student’s t-test calculated over the 21 subjects, and (2) analysis of variance (ANOVA) to investigate whether there were any statistically significant differences between the brain responses to different emotions. ANOVA assumes that (1) the variances across conditions are equal (correct for our data), (2) the population distribution is approximately normal (we observe close to normal distribution in responses when investigating areas of interest) and (3) all samples are drawn independently from each other (the subjects were measured in separate sessions and we can assume the brains function independently of each other). Bartlett’s test was used to ensure that the normality and equal variance assumptions were not violated. If ANOVA rejected the null hypothesis that all conditions come from the same mean, Tukey-Kramer post hoc test was used to determine which conditions differ from each other significantly, if any. Finally, we tested each of the stimuli separately to see whether there is a response that differs statistically significantly from zero.

#### Voxel-based clustering analysis

The image regions corresponding to the white and gray brain matter were smoothed with a Gaussian filter of radius 1.5 voxels to reduce the effect of noise on the voxel-based clustering. To find the location of regions that show the greatest statistical significance and to estimate the extent of the phenomena, we use adaptive voxel-based clustering^5^. The voxel-wise statistical significance is thresholded with an initial value of p_th,1_ = 0.001 and contiguous regions of voxels with p < p_th,1_ are identified as potential clusters. In the next step, each region is expanded to include neighboring voxels that pass the less stringent tests p < p_th,2_ = 0.0033 and p < p_th,2_ = 0.01. Regions which are separate at the higher significance level but merge at a lower significance levels are considered one cluster. The cluster p-value is calculated by averaging the HbT values for voxels within the cluster for each of the three significance levels, calculating the statistical test at the group level (either Student’s t test or ANOVA) for each voxel-wise significance level and selecting the level which produces the smallest p-value as the final cluster-wise p-value. In Fig. 3 that illustrates the cluster locations and extents, voxel-wise p-values of 0.001 and 0.01 are shown to illustrate how the cluster extent depends on the threshold. The purpose of averaging the voxels in the cluster is to reduce the effect of noise and improve statistical significance. Finally, to minimize the occurrence of spurious clusters which can appear near the edges of the FOV, we required that each cluster reported includes at least 200 voxels. A genuine activation should reconstruct into a larger volume due to the diffuse nature of the imaging method and smaller clusters are likely to be artifacts. The cluster p-values were then subjected to correction for multiple comparisons considering the practically separately imageable regions that the method is capable of distinguishing. The Bonferroni method was used to correct for multiple comparisons. The correction factor was estimated by considering that the imaging method is able to image distinct features of approximately 1 cm^3^ in volume within the FOV which has a gray matter volume of 80 cm^3^, leading to N_MC,1_ = 80. A second estimate was derived by considering the average number of source-detector pairs in use with SDS > 12 mm, which was N_MC,2_ = 120. We chose the larger number N_MC_ = 120 to minimize the probability of unwanted false positives.

The statistical tests that were considered for the voxel-based clustering were: (1) comparison between the average across all conditions and zero using Student’s t-test, (2) comparison between the different conditions using ANOVA, and (3) comparison between each of the stimulus conditions with baseline averaged within the window from −1 s to 0 s relative to stimulus train onset.

#### Region of interest (ROI) –based analysis

We identified six regions of interest (ROIs) on the left hemisphere for our study (Fig. 4): (i) Located in the anterior superior temporal sulcus (aSTS), (ii) in the superior temporal gyrus (STG), (iii) in the inferior frontal gyrus (IFG), (iv) in the left anterior insula (AI), (v) in the mid-insula (MI), and (vi) in the posterior STS (pSTS). These ROIs were selected based on previous studies showing activation in these brain regions in adults during speech perception and emotional processing. Specifically, left STS is involved in speech perception^92–94^ and the STG is the site of auditory association cortex (and a site of multisensory integration) and is activated during both speech and sound processing^93,95–97^. The IFG is involved in multiple aspects of word recognition, including both semantic and phonological processing^58,97^. Left insular (AI) activation has been suggested as an effect of selectively attending to the vocal stimuli^98,99^. Left dorsal mid-insula (MI) is implicated in speech perception^95,100^. Left pSTS plays an important role in the learning and neural representation of unfamiliar sounds^101^.

## Supplementary information

Supplement - Time courses for the regions of interest

## Data Availability

Data recorded and analysed in the study are available upon contacting the corresponding author with a reasonable request. The data sharing will be subject to the limitations specified in the consent form and Finnish law.
