# Supplementary material for: Hemodynamic responses to emotional speech in two-month-old infants imaged using diffuse optical tomography
Source: Sci Rep. 2019 Mar 18;9:4745. doi: 10.1038/s41598-019-39993-7 (PMC6426868; doi:10.1038/s41598-019-39993-7)
Supplement: Supplementary file 1 — Supplement - Time courses for the regions of interest [file 41598_2019_39993_MOESM1_ESM.docx]

**Hemodynamic responses to emotional speech in two-month-old infants imaged using diffuse optical tomography**

Shashank Shekhar^1,2^, Ambika Maria^1^, Kalle Kotilahti^3^, Minna Huotilainen^1,4,5^, Juha Heiskala^6^, Jetro J. Tuulari^1^, Pauliina Hirvi^3^, Linnea Karlsson^1,7^, Hasse Karlsson^1,8^, and Ilkka Nissilä^3^

^1^ University of Turku, Institute of Clinical Medicine, Turku Brain and Mind Center, FinnBrain Birth Cohort Study

^2^ University of Mississippi Medical Center, Department of Neurology, MS, USA

^3^ Department of Neuroscience and Biomedical Engineering, Aalto University, Finland

^4^ CICERO Learning, Faculty of Educational Sciences, University of Helsinki, Finland

^5^ Faculty of Educational Sciences, University of Helsinki, Finland
^6^ Department of Clinical Neurophysiology, Helsinki University Central Hospital, Finland
^7^University of Turku and Turku University Hospital, Department of Child Psychiatry
^8^ University of Turku and Turku University Hospital, Department of Psychiatry


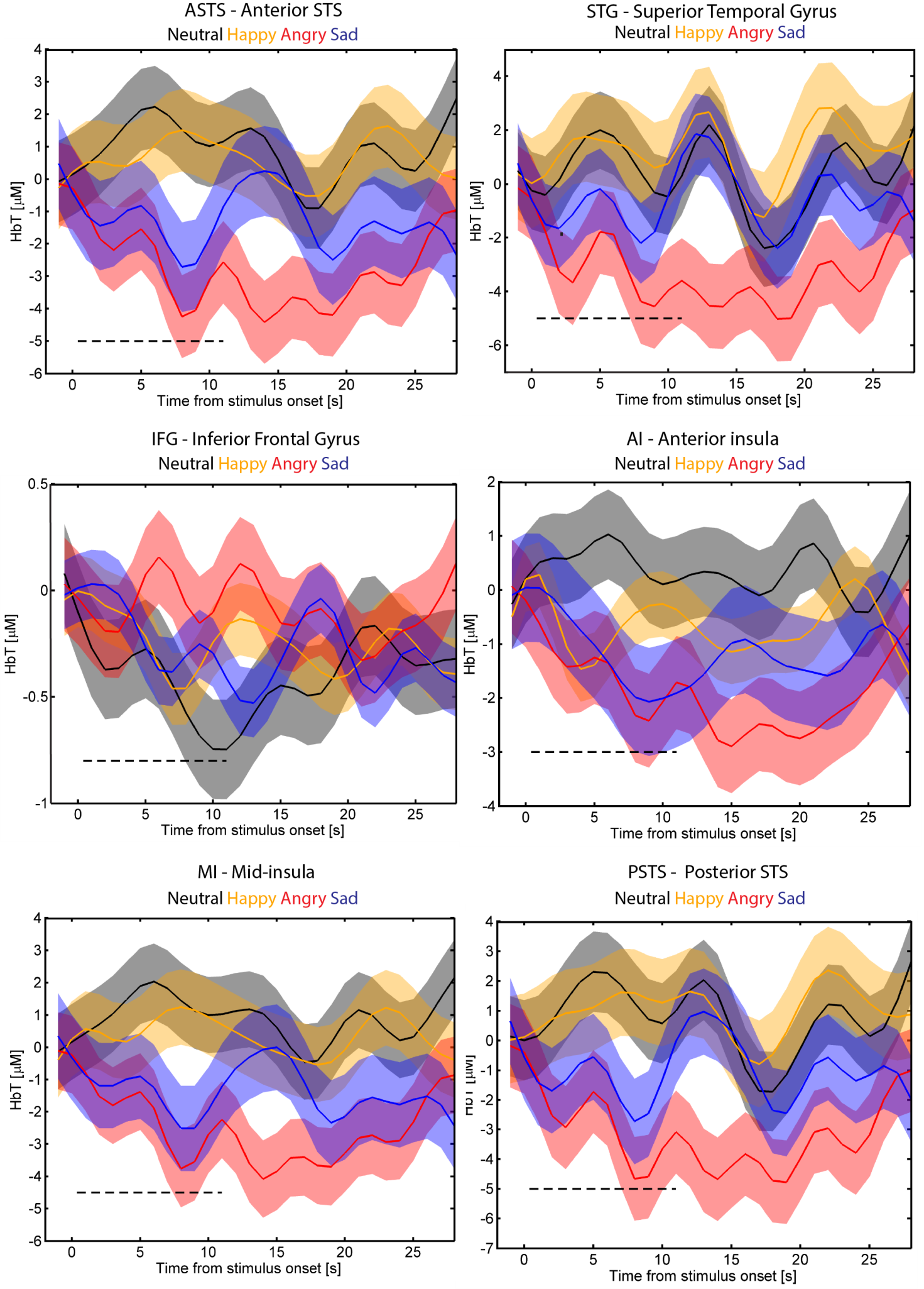


*Figure S1. Time courses of the HbT responses to emotional speech in the six ROIs. The stimulus presentation is indicated with a horizontal dashed black line.*
